# Editorial and Miscellaneous

**Published:** 1861-05

**Authors:** 


*Death of David Meredith Reese, M. D. LL. D.*—This widely known and distinguished medical practitioner, teacher, author and editor, died in New York city the 20th inst., if the daily press brings correct information. We were somewhat prepared for this event, by the descriptions of his case that have been given in the medical press during the past season. A correct memorial of his life, it is to be hoped, will speedily be published. With very considerable attainment, he combined a versatility of talent and readiness of adaptation which rendered him peculiarly formidable in that field for which his faculties best fitted him, and wherein he won his highest laurels—controversy. Dr. Reese scarcely, if ever, brought the lance to rest which once he had couched, or quit the field whilst an enemy remained upon it. All his life long he honored and sustained the profession in that position which he believed it ought to occupy. If in sustaining his occasionally erratic views, he came into collision with any one, all must admit he was an open and honorable foe; he scorned bush fighting or evasion of any sort. With quick coming flashes of the fiery and impetuous, he combined withal a really genial and easily conciliated nature. This not unfrequently led him into strange inconsistencies, but those who knew him best will coincide with the remark that the source of many of these was such as really to do him honor.

The writer of the present notice, a score of years since, knew and respected him, has ever been on personally friendly relations with him, and yet has been frequently compelled to enter the lists against him in earnest contest. The narrow cliques which so largely influence professional life in New York city, could not discourage or rebuff him; he carved himself a place and fame among them, and established his widely disseminated journal, the *American Medical Gazette*, by his individual efforts when a mortal epidemy seemed to sweep away one after another of those sustained by large *quasi* associations.

That Dr. Reese was free from several objectionable characteristics, his best friends will not claim; that he has accomplished much for the welfare and advancement of the profession, all must admit. All his life-long he was a *live* man, and a working man. We know many a knavish, plotting, scheming, intriguing charlatan, who will breathe freer, and stride along with a loftier air, now that his keen and trenchant blade is forever sheathed by death; but among the good men and true of our ranks, we do know that there has been and will be a sensation of unfeigned sorrow as the painful intelligence is received.

Truly, "after life's fitful fever, he sleeps well," for he rests beneath memories which bend lovingly over him, as the cypresses and willows over his tomb in beautiful Greenwood.

*Appointment.*—Ralph N. Isham, M. D., has been appointed to the position of Surgeon to the Chicago Marine Hospital. We learn from a contemporary that this appointment gives satisfaction to not only the gentleman himself who has drawn the prize, but to his friends and admirers. From the same source we learn that "Dr. Isham is a gentleman whose professional attainments, pleasing address, and brilliant style of operating, have won for him no mean reputation, both in the amphitheater and in private practice."

---

*Gen. McLellan.*—Perhaps some of our readers may not be aware that this eminent gentleman, to whom has been assigned the command of the North-western division of the national forces, is a son of the late distinguished Prof. George McLellan of Philadelphia, founder of the Jefferson University Medical College. Under his supervision there can be no doubt that the most enlightened and sagacious measures will be adopted for securing the best sanitary condition of the army.

---

*New Practical Treatise on Military Surgery.*—Bailliere & Brothers, 440 Broadway, New York City, announce as forthcoming, in a few days, a new work on this subject, by Prof. Frank H. Hamilton, M. D., the well known author and lecturer. The book cannot fail of being valuable and interesting, as it is compendious and timely. It will be in one vol. octavo, and will be mailed, postage free, on receipt of $2.00, sent to their address.

---

*Financial.*—Will our subscribers please to remember, in forwarding their subscriptions, that the large majority of bills now in circulation are ruinously below par? We will receive at par, on subscriptions, bills on all specie paying banks, and on all banks based on the stocks of Northern States or the United States. For other bills we can only credit their market value. All business men will appreciate this proposition as being as liberal as could be desired during the present currency storm.

